# The effects of cognitive behavioral therapy-based psychoeducation on quality of life, anxiety, and depression in children with attention deficit hyperactivity disorder

**DOI:** 10.1186/s40359-026-04110-7

**Published:** 2026-02-07

**Authors:** Vedat Argın, Zerrin Çigdem, Hatice Altun, Adem Doğaner

**Affiliations:** 1 Vocational School of Health Services, First and Emergency Aid Program, Kahramanmaraş Sütçü İmam University, Kahramanmaraş, Turkey; 2 Faculty of Health Sciences, Department of Nursing, Istanbul Topkapi University, Istanbul, Turkey; 3 Faculty of Medicine, Department of Child Psychiatry, Kahramanmaraş Sütçü İmam University, Kahramanmaraş, Turkey; 4 Faculty of Medicine, Department of Biostatistics and Medical Informatics, Kahramanmaraş Sütçü İmam University, Kahramanmaraş, Turkey

**Keywords:** Cognitive behavioral therapy, Quality of life, Anxiety, Depressive disorder, Attention deficit disorder with hyperactivity

## Abstract

**Background:**

Attention deficit hyperactivity disorder is a prevalent neurodevelopmental condition in childhood, associated not only with difficulties in attention and impulsivity but also with increased internalizing symptoms, such as anxiety and depression, and a marked reduction in quality of life. Although pharmacological treatments play a central role in the management of attention deficit hyperactivity disorder, there is a growing need for psychosocial interventions that target these emotional difficulties and quality of life outcomes while actively involving families. This study is novel in its evaluation of the effectiveness of a structured cognitive behavioral therapy based psychoeducational program involving both children and their parents in reducing anxiety and depressive symptoms and improving quality of life in children diagnosed with attention deficit hyperactivity disorder.

**Method:**

A quasi-experimental controlled pretest–posttest design was employed. The intervention group comprised thirty-seven children aged eight to twelve years diagnosed with attention deficit hyperactivity disorder and their parents, while the control group included thirty-six age- and gender-matched children with the same diagnosis and their parents. The intervention consisted of an eight-session cognitive behavioral therapy based psychoeducational program integrating emotional regulation, adaptive coping strategies, and parent-focused guidance. Data were collected at baseline and after the intervention using a sociodemographic information form, the Quality of Life Inventory, and the Revised Children’s Anxiety and Depression Scale.

**Results:**

The intervention and control groups were similar in terms of demographic characteristics. The mean age of children in the intervention group was 10.35 ± 1.40 years, while the mean age of children in the control group was 10.47 ± 1.28 years (*p* = 0.751). Regarding gender distribution, the intervention group consisted of 11 girls (29.7%) and 26 boys (70.3%), while the control group consisted of 18 girls (50.0%) and 18 boys (50.0%) (*p* = 0.077). Children participating in the cognitive behavioral therapy -based psycho-educational program showed a statistically significant improvement in quality of life and a significant decrease in anxiety and depressive symptoms between the pre-test and post-test. No significant changes were observed in the same outcome measures in the control group.

**Conclusion:**

These findings provide robust evidence that cognitive behavioral therapy based psychoeducational interventions incorporating family participation are effective in improving psychological well-being and quality of life in children with attention deficit hyperactivity disorder. From both psychiatric and nursing perspectives, such programs represent a valuable, non-pharmacological adjunct to standard care, supporting holistic and family-centered approaches in the multidisciplinary management of childhood attention deficit hyperactivity disorder.

**Clinical trial registration:**

This study was registered under Clinical Trial Number: NCT06624527 on September 25, 2023. URL: https://clinicaltrials.gov/study/NCT06624527?term=NCT06624527&rank=1.

**Supplementary Information:**

The online version contains supplementary material available at 10.1186/s40359-026-04110-7.

## Introduction

Attention deficit hyperactivity disorder (ADHD) is a neurodevelopmental disorder commonly observed in childhood, characterized not only by difficulties in attention and impulsivity but also by increased internalizing symptoms such as anxiety and depression, as well as a reduction in quality of life [1]. ADHD is a highly prevalent disorder worldwide, with prevalence rates varying according to age, diagnostic criteria, and assessment methods [2]. In Turkey, a study conducted by Şenol et al. (2018) evaluated 1,024 school-aged children between 7 and 15 years old and reported an ADHD prevalence of 6.2%, highlighting the public health significance of ADHD among the Turkish pediatric population [3]. In contrast, an epidemiological study conducted in Turkey by Öztürk et al. (2025) examined 689 preschool children aged 3 to 6 years and found an ADHD prevalence of 3.3%, indicating that ADHD can also be present during early childhood [4].

Emerging from the complex interaction of genetic, environmental, and biological factors, ADHD leads to significant impairments in family, school, and social functioning. Children with ADHD frequently experience academic underachievement, low self-esteem, emotional and behavioral difficulties, and comorbid psychiatric conditions such as anxiety and depression [5–7]. Although symptoms typically appear in childhood, they may persist into adulthood, increasing the risk of comorbidities such as substance use disorders and anxiety- or depression-related conditions [8]. Consequently, early and effective intervention is critical to prevent the long-term psychiatric, interpersonal, and familial consequences of ADHD [8, 9].

While pharmacological treatment is generally considered the first-line approach for ADHD, existing evidence indicates that medication alone is insufficient to address the complex psychosocial dimensions of the disorder [10]. Current literature has limitedly examined the effects of parent-inclusive cognitive-behavioral therapy (CBT)-based psychoeducational programs on children’s functional outcomes, psychosocial well-being, and quality of life. Despite growing evidence supporting CBT and psychoeducation in ADHD, a notable gap remains regarding the specific impact of parent-inclusive CBT-based psychoeducational programs on children’s psychosocial functioning and quality of life. This study directly addresses this gap by evaluating a structured, family-centered CBT-based intervention aimed at enhancing both child outcomes and parental involvement, thereby underscoring the need for research that informs and improves current treatment strategies for children with ADHD.

Existing studies have predominantly focused on child-centered interventions, with limited investigation into the effects of parent-inclusive CBT-based psychoeducation programs on children’s functioning, psychosocial well-being, and quality of life [1, 9]. The sustainable effects of family-centered approaches and the generalization of therapeutic gains to the home environment remain underexplored. This study emphasizes the importance of parental involvement in ADHD treatment, providing evidence to guide clinical practice and contribute to the improvement of current treatment strategies [1, 9].

In this context, psychosocial interventions based on psychoeducation and CBT have gained increasing prominence [10, 11]. Conceptually, psychoeducation and CBT are complementary approaches: psychoeducation equips children and families with fundamental knowledge and awareness about the disorder, while CBT builds upon this understanding by offering structured techniques for cognitive and behavioral change. Together, these approaches function synergistically to promote adaptive coping, emotional regulation, and improved daily functioning [12, 13]. Therefore, psychoeducation and CBT are considered theoretically and clinically complementary approaches that support cognitive, emotional, and behavioral change in the management of ADHD [5, 13–17].

Psychoeducation not only enhances treatment adherence but also helps children manage stress more effectively and improve overall quality of life. CBT, on the other hand, is effective in managing impulsivity, behavioral problems, and comorbid anxiety and depression in children with ADHD [5, 13–17]. Within this multidisciplinary framework, pediatric nurses play a crucial role in implementing CBT-based psychoeducational programs, guiding families, and actively participating in the treatment process, thereby promoting both treatment adherence and psychosocial well-being [12, 13].

Moreover, involving parents directly in CBT-based psychoeducation represents an innovative approach compared with previous studies, which have mostly focused solely on children [18, 19]. Parental involvement is both theoretically and clinically significant, as it facilitates the generalization of therapeutic gains to the home environment, strengthens parent–child relationships, and reinforces consistent behavioral patterns that sustain treatment outcomes. Unlike prior research, this study emphasizes a family-centered model that acknowledges parents as active agents in the therapeutic process, extending the scope of CBT-based psychoeducation beyond the individual child.

Despite substantial evidence supporting the efficacy of CBT and psychoeducation in ADHD, research specifically examining the effects of parent-inclusive CBT-based psychoeducational programs on children’s psychosocial functioning, quality of life, and emotional well-being is limited. By addressing this gap, the present study aims to provide novel insights into how family involvement enhances the sustainability and overall impact of therapeutic outcomes.

In summary, alongside pharmacological strategies, CBT-based psychoeducational interventions constitute an essential component of ADHD treatment. Such programs have the potential to improve the quality of life and psychological well-being of children with ADHD and their families. Building on this theoretical and empirical framework, the primary aim of this study was to investigate the effects of a CBT-based psychoeducational program designed to reduce functional impairments and enhance coping skills on the quality of life, anxiety, and depression levels of children with ADHD.

## Materials and methods

### Study design

This study was designed as a quasi-experimental controlled study utilizing a pretest–posttest design. In this design, participants were unaware of their group allocation (intervention or control.

### Research hypotheses

- Hypothesis 1 (H1): CBT-based psychoeducation programs affect the quality of life of children with ADHD.
- Hypothesis 2 (H2): The CBT-based psychoeducation program affects the anxiety and depression levels of children with ADHD.

### Study setting and duration

The study was conducted at the Child Psychiatry Department of a university hospital in Turkey between September 25, 2023, and August 29, 2024.

### Ethical considerations

Ethical approval was obtained from the Ethics Committee of University A (Approval No: 2023/31, Date: 31.01.2023). The study adhered to the Declaration of Helsinki and relevant ethical guidelines. Institutional permissions were secured prior to the research. The study was registered with Clinical Research Number NCT06624527 and was funded by the Scientific and Technological Research Council of Turkey (TÜBİTAK) under project number 323S232. All the facilitators received structured training on the psychoeducational protocol (Barcode No: UN_041054323475554869027), and necessary permission for all the measurement tools was obtained. Informed consent was secured from all participants. Ethical integrity was ensured by offering the CBT-based psychoeducation program to the control group after August 29, 2024.

### Population and sample

The study population consisted of children with ADHD and their parents who presented to the Child Psychiatry Department of a university hospital in Turkey. Participants were selected based on predefined inclusion criteria and voluntary participation. Specifically, a convenience sampling method was employed, whereby eligible children and their parents presenting during the study period were invited to participate. This approach allowed for practical recruitment within the available population while maintaining adherence to inclusion and exclusion criteria.

The sample size was determined prior to the initiation of the study based on reference results from previous research in the literature [18, 19]. According to the data entered into the G*Power 3.1.9.7 software, with an alpha level of 0.05, an effect size of 0.8, and a 95% confidence interval, the required number of participants for each group was calculated as 35 children and their parents in both the intervention and control groups. Each group originally included 35 children and their parents, and this number was increased to 40 per group to account for potential dropouts and enhance statistical power.

The inclusion criteria for children participating in the study were as follows: having a diagnosis of ADHD according to DSM-5 diagnostic criteria; absence of psychiatric and physical comorbidities accompanying ADHD; no diagnosis of intellectual disability; not receiving pharmacological treatment; being aged 8 to 12; being literate; having the ability to communicate; and being willing to cooperate.

The inclusion criteria for parents participating in the study were as follows: providing consent for their child’s participation; living with the child; being the biological parent or primary caregiver (first-degree guardian) of the child; absence of hearing or speech impairments; no diagnosed or treated psychiatric disorders; ability to communicate; and openness to cooperation. Children or parents who did not meet the specified criteria were excluded from the study and evaluated according to the exclusion criteria.

### Data collection instruments

Data were collected using the *Personal Information Form*, which was developed based on the relevant literature for both children and their parents [20–22], along with three standardized measurement tools: the *Pediatric Quality of Life Inventory (PedsQL)*, the *Revised Child Anxiety and Depression Scale–Child Version (RCADS-CV)*, and the *Parent Version (RCADS-PV)*.

The Pediatric Quality of Life Inventory (PedsQL), developed by Varni et al. (1999), is a 23-item instrument designed to assess health-related quality of life in children aged 2–18 years across five subdomains [23]. The scale provides three main scores: the Total Scale Score, the Physical Health Summary Score, and the Psychosocial Health Summary Score, which includes emotional, social, and school functioning domains. The Turkish adaptation of the scale was conducted by Memik et al. (2007) [24].

The Revised Child Anxiety and Depression Scale (RCADS), developed by Chorpita et al. (2000), is a standardized instrument used to assess anxiety and depression symptoms in children [25]. The scale includes child and parent forms and consists of 47 items across six subscales: Separation Anxiety, Social Phobia, Obsessive-Compulsive Disorder, Panic Disorder, Generalized Anxiety Disorder, and Major Depressive Disorder. The instrument yields six subscale scores, a Total Anxiety Score, and a Total Anxiety-Depression Score, calculated based on the child’s age and gender using the RCADS-PV scoring program developed by the original authors. The Turkish adaptation of the RCADS was conducted by Görmez et al. (2017) [26].

Within the context of this study, the PedsQL and RCADS scales were used to assess health-related quality of life and anxiety–depression symptoms, respectively.

### Data collection procedure

After ethical approval and institutional permissions were secured, data collection was carried out in two main phases:

### Phase 1: program development

Prior to the study, the researcher participated in a practical training program on CBT applications at a higher education institution. Following this, the CBT-based psychoeducational program was systematically developed based on evidence-based guidelines derived from the literature [27–29]. The structure, content, and session components of the program were determined through a combination of literature review and expert consultation.

The program was reviewed by six academics with expertise in child health and extensive experience in implementing cognitive-behavioral therapy interventions. These experts evaluated the program in terms of content coherence, session duration, and developmental and clinical appropriateness. Based on their feedback, necessary revisions were made to ensure alignment with core therapeutic goals and to enhance the feasibility of the sessions for both children and parents. Based on their feedback, necessary adjustments were made to ensure that each session addressed core therapeutic goals while remaining age-appropriate and applicable for both children and their parents. All sessions were conducted individualy. The content of the CBT-based psychoeducational program was structured around the following topics: The first session of the psychoeducational process included an introduction and cognitive behavioral therapy-based debriefing. The second session addressed ADHD, and the third session addressed emotions. Impulsivity and hyperactivity were addressed in the fourth session, control strategies were taught in the fifth session, attention development was addressed in the sixth session, appropriate behaviors were selected in the seventh session, and the eighth session ended with a general evaluation (see Supplementary Material 1).

### Phase 2: assignment procedure

Children with ADHD who presented to the Child Psychiatry outpatient clinic and their parents were included in the study. The sample group was formed by obtaining contact information from children and parents who had been diagnosed by a specialist child psychiatrist and who agreed to participate in the study.

Participants were assigned to two groups (intervention or control) using a predetermined odd/even numbering rule; no centralized or concealed allocation methods were applied. This allocation method may introduce selection bias; for instance, the investigator or participants could anticipate the assignment sequence, potentially causing baseline differences between groups. This potential bias represents an important limitation and was considered in the interpretation of the study results. The samples were sequentially numbered and assigned to two groups: odd numbers were allocated to the intervention group, and even numbers to the control group. The principal investigator had access to the allocation sequence, while the team responsible for enrolling participants and conducting assessments was not informed of group assignments in advance.

However, the facilitators and outcome assessors could not be fully blinded. Partial blinding was applied: the team responsible for participant enrollment and assessments was unaware of group allocation. However, facilitators delivering the intervention and outcome assessors could not be blinded, which may have introduced bias in subjective outcome measures. This potential source of bias was acknowledged and taken into account when interpreting the results. Therefore, blinding was limited to the team responsible for participant enrollment and assessment, and full blinding could not be ensured for those delivering the intervention or evaluating the outcomes.

Participants were informed about the study. A therapy-friendly room was prepared in the clinic for data collection, and parents were contacted by phone to schedule convenient dates and times. Participants (children and their parents) were not informed of their group assignment. All were told they would receive CBT-based psychoeducation. However, only the intervention group received CBT-based psychoeducation during the study. The control group received CBT-based psychoeducation after the study to avoid any ethical violations.Due to the structural differences in the intervention, blinding of facilitators and outcome assessors was not possible. This limitation was acknowledged as a constraint of the study.

The first two sessions of the CBT-based psychoeducation program were conducted for both children and parents. Subsequent sessions were conducted with children only, but parents participated as observers. At the end of each session, the practices were explained and taught to parents, and worksheets were provided as homework so that similar exercises could be continued at home. Furthermore, parents were provided with the necessary information and guidance on how to support and monitor their children during the homework process. This approach ensured the simultaneous and active participation of both children and parents in all sessions.

A notable and unique aspect of this study is the simultaneous organization of CBT-based psychoeducation sessions for both children and parents. This holistic approach aims to facilitate parents’ active participation in the therapy process, increase the retention of children’s gains, and support them in the home environment. This implementation model differs from similar studies in the literature and makes a unique contribution to the field.

### Data collection from the control group

**Fig. 1:**
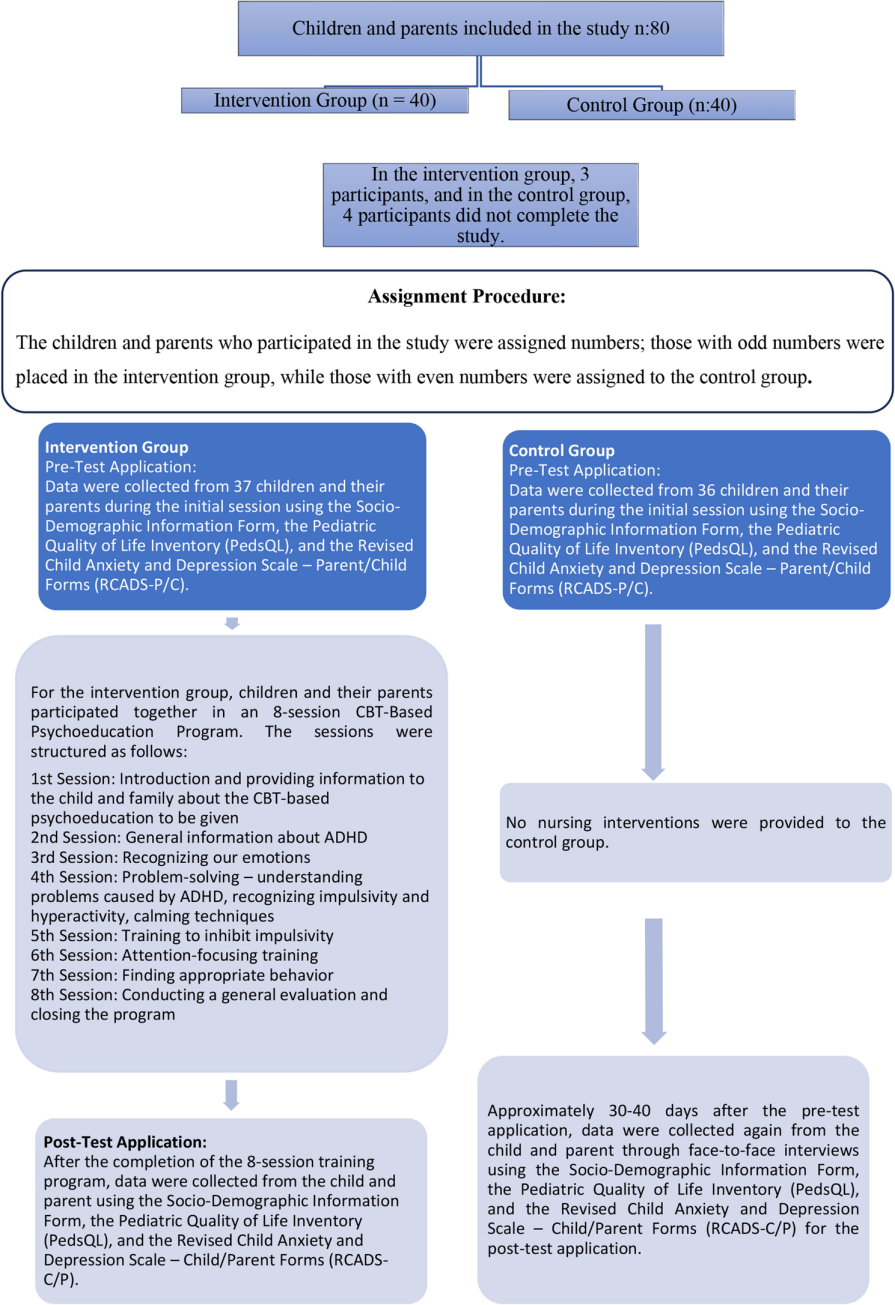
Research flowchart

Pretest assessments for the control group were conducted via face-to-face interviews lasting approximately 20–25 min. Posttest assessments were conducted in the intervention group, coinciding with the last session of the CBT-based psychoeducation program, and in the control group, approximately 30–40 days later during routine clinic visits. This small posttest time difference was considered a limitation of the study (Fig. 1).

### Data collection in the intervention group

The intervention group participated in eight weekly face-to-face sessions, each lasting 40–45 min, a duration determined to be appropriate for the 8–12 age group as it aligns with CBT-based interventions for similar populations and is supported by literature and expert opinions considering children’s attention span, cognitive capacity, and engagement level; accordingly, the psychoeducation content was structured to match participants’ developmental characteristics and enriched with games, visual materials, and interactive activities, making the 40–45-minute duration suitable [19, 30]. Accordingly, the psychoeducation content was structured to match participants’ developmental characteristics and enriched with games, visual materials, and interactive activities, rendering the 40–45-minute session length appropriate. Pretests were administered during the first session, and posttests during the final (eighth) session. Interviews outside of session activities lasted 20–25 min.

Both parents participated in the pre- and post-intervention assessments. In cases where both parents were present, it was recommended that the parent completing the questionnaire be the one with more daily interaction with the child; therefore, in line with common practices in the literature, the mother was prioritized [19, 26]. An overview of the research methodology is presented in the flow chart shown in Fig. 1.

During the study, three participants from the intervention group and four from the control group withdrew due to missing data or personal reasons. Consequently, data were collected from 37 children and parents in the intervention group and 36 children and parents in the control group.

### Assessment of harms

Potential harms or adverse effects were assessed using an open-ended evaluation approach. At the end of each session, both children and their parents were asked whether they had experienced any discomfort, stress, emotional difficulty, or negative experiences. Participants were also allowed to report any undesirable situations at any time during the study. Serious harm or emotional distress was operationalized as cases in which children displayed marked anxiety, sadness, behavioral changes, or reported feelings of insecurity. Any reported adverse events were documented, and when necessary, a child psychiatrist was consulted to provide appropriate intervention. No serious harm or adverse effects were reported throughout the study period.

### Interim analyses and stopping guidelines

No interim analyses were planned or conducted, and no predefined stopping criteria or guidelines were applied during the study. No harms or adverse events were observed or reported in either group during the study period.

### Cognitive-behavioral therapy based psychoeducation program

The program was structured as an 8-session CBT-based psychoeducation intervention. Each session applied cognitive, behavioral, and emotional regulation techniques according to predefined objectives. The sessions addressed topics including introduction and orientation, ADHD awareness, emotion recognition, problem-solving, impulse control, attention focusing, appropriate behavior selection, and overall program evaluation. Methods included the ABC model, role-playing, problem-solving steps, attention-focusing exercises, emotion cards, and visual/multimedia materials. Homework assignments reinforced acquired skills, and parents were provided with informational materials. The therapist was certified in CBT and ADHD interventions. Program fidelity was systematically monitored through structured checklists completed by independent and trained evaluators on selected sessions. Regular supervision by a field expert ensured that any deviations were promptly addressed. Sessions were adapted to be developmentally appropriate. This rigorous monitoring and evaluation process ensured that the program’s objectives, technical features, and implementation procedures were clearly and transparently presented (see Supplementary Material 1).

### Data analysis

The analyses were primarily conducted on the subdimensions of the scale; however, the total scale scores were also included in the analyses and reported. No additional subgroup or sensitivity analyses were performed beyond the pre-specified primary analyses. The normality of the quantitative variables was assessed via the Shapiro–Wilk test. Differences between the intervention and control groups were analyzed via the independent samples t test for variables meeting the normality assumption. For variables that violated this assumption, group comparisons were conducted via the Mann–Whitney U test. To evaluate differences between pretest and posttest measurements, the paired t test was applied for normally distributed variables, whereas the Wilcoxon signed-rank test was employed for variables that were not normally distributed. Distributional differences between groups for categorical variables were examined via the chi-square test and Fisher’s exact test, whereas repeated measures of categorical variables were assessed via the McNemar test. Relationships between quantitative variables were investigated via Spearman’s correlation analysis. The reliability of the scales was determined by calculating Cronbach’s alpha coefficients. Statistical parameters are reported as the means, standard deviations, medians, percentages (%), and frequencies (n). Missing data were handled using listwise deletion (complete case analysis); given the relatively small sample size, this approach may have reduced statistical power, and this limitation is acknowledged. A significance level of *p* < 0.05 was set for all analyses. Data analyses were performed via IBM SPSS Version 22, the RCADS-PV parent scoring program version 32 (Excel), and Jamovi 2.3.16 software.

## Results

No statistically significant differences were detected between the intervention and control groups in terms of mean age, gender distribution, educational status, or self-assessment of academic performance (*p* > 0.05). A statistically significant difference was identified between groups regarding the presence of hobbies (*p* = 0.001, χ²=19.802) (*p* < 0.05). Furthermore, a significant difference was found between groups concerning the degree of parental closeness to the child (*p* = 0.021, U = 495.500) (*p* < 0.05). The sociodemographic characteristics of the children and their parents included in the study are presented in the Supplemental Files (Table 1). The similarity of sociodemographic characteristics between the intervention and control groups is critical for accurately assessing the effects of the CBT-based psychoeducation program administered to the intervention group (Table 1).

**Table 1:** Comparison of the sociodemographic variables of children and their parents across groups

Variables	Intervention Group (*n* = 37)		Control Group (*n* = 36)		*p*	Test Statistic
	Ort ± SS		Ort ± SS			
Child Mean Age	10.35 ± 1.40		10.47 ± 1.28		0.751	x^2^=0.951
Parent Mean Age	38.92 ± 5.53		40.19 ± 5.07		0.159	x^2^=2.675
	**n**	**%**	**n**	**%**		
Child Gender						
Female	11	29.73	18	50.00	0.077	x^2^=3.131
Male	26	70.27	18	50.00		
Child Educational Level						
Primary School	22	59.46	24	66.67	0.630	x^2^=1.342
Middle School	15	40.54	12	33.33		
Self-Evaluation of School Performance						
Good	16	43.24	10	27.78	0.112	U = 502.000
Average	16	43.24	14	38.89		
Poor	5	13.52	12	33.33		
Having a Hobby						
Yes	24	64.86	5	13.89	0.001*	**x** ^**2**^ **=19.802**
No	13	35.14	31	86.11		
Primary Caregiver						
Mother	29	78.38	19	52.78	0.021*	**U = 495.500**
Father	8	21.62	17	47.22		
Parent Educational Level						
Literate	7	18.92	1	2.77	0.094	x^2^=7.759
Primary Education	9	24.32	18	50.00		
High School	9	24.32	6	16.67		
Higher Education	11	29.74	9	25.00		
Postgraduate	1	2.70	2	5.56		
Parent Research Experience						
Yes	19	51.35	24	66.67	0.236	x^2^=1.768
No	18	48.65	12	33.33		
Family Type						
Nuclear Family	28	75.68	25	69.44	0.472	U = 615.500
Single Parent or Divorced	5	13.51	4	11.12		
Extended Family	4	10.81	7	19.44		
Family Income Level						
Income Higher than Expenses	14	37.84	9	25.00	0.200	U = 563.500
Income Equal to Expenses	20	54.05	22	61.11		
Income Lower than Expenses	3	8.11	5	13.89		
Parent’s Evaluation of Child’s School Performance						
Good	12	32.43	5	13.89	0.054	U = 523.500
Average	23	62.16	27	75.00		
Poor	2	5.41	4	11.11		
Duration Since Child’s ADHD Diagnosis						
0–6 months	4	10.81	3	8.33	0.897	U = 655.000
7–12 months	3	8.11	4	11.11		
13–24 months	9	24.32	8	22.22		
25 months or more	21	56.76	21	58.34		
Parent’s Prior Knowledge of ADHD Diagnosis						
Yes	26	70.27	28	77.78	0.595	x^2^=0.534
No	11	29.73	8	22.22		
Presence of Diagnosed Children with ADHD in Environment (Sibling/Cousin)						
Yes	23	62.16	18	50.00	0.295	x^2^=1.096
No	14	37.84	18	50.00		
Child’s ADHD Therapy Status						
Yes	7	18.92	12	33.33	0.163	U = 570.000
No	30	81.08	24	66.67		
*Significant at *p* < 0.05						

In addition to the subscale analyses described in the Methods section, total scores were also calculated and analyzed. According to the findings presented in Table 2, significant differences were observed in various subscales of the PedsQL. For the physical health subscale, significant differences were found between the pretest and posttest scores in both the intervention and control groups, with the intervention group showing (*p* < 0.001, U = 436.000), and the control group showing (*p* < 0.001, U = 217.000). For the emotional functioning subscale, a significant increase in posttest scores was observed in the intervention group (*p* < 0.001, U = 237.000), whereas no significant difference was found in the control group. Similarly, in the social functioning subscale, the intervention group demonstrated a significant increase in posttest scores (*p* < 0.001, U = 325.500), whereas no significant difference was detected in the control group. With respect to the School Functioning subscale, a significant increase was observed in the intervention group’s posttest scores (*p* < 0.001, U = 278.500), but the scores of the control group did not significantly change. For the Psychosocial Health subscale, a significant increase in posttest scores was found in the intervention group (*p* < 0.001, t = 3.241). Finally, with respect to the total PedsQL scores, the intervention group presented a significant increase in posttest scores (*p* < 0.001, U = 202.500), whereas the control group did not show a significant difference. Within-group analyses revealed significant differences in the intervention group (*p* < 0.001), but no significant differences were detected in the control group (*p* > 0.05). In conclusion, the intervention group showed marked improvements following treatment, whereas no changes were observed in the control group (Table 2).

**Table 2:** Comparison of the pretest and posttest subscale and total scores of the children’s ADHD rating scale between and within groups

PedsQL Rating Scale Subscales and Total Scores (Pre-Test and Post-Test Assessment)	Intervention Group		Control Group			
	Median	Ort ± Ss	Median	Ort ± Ss	*p*	Test Statistic
Pre-Test Application; Physical Health Subscaleᵇ	75.00	65.29 ± 23.91	51.56	58.82 ± 19.24	**p** < 0.001*	**U = 436.000**
*Post-Test Application; Physical Health Subscaleᵇ	85.00	82.30 ± 14.22	65.00	62.92 ± 12.27	***p*** < 0.001*	**U = 217.000**
p value and Test Statisticᵈ	***p*** < 0.001* Z:-3.490		0.004* Z:-2.905			
Pretest Application; Emotional Functioning Subscaleᵇ	65.00	61.62 ± 18.82	60.00	61.81 ± 12.94	0.781	U = 612.000
Posttest Application; Emotional Functioning Subscaleᵇ	85.00	84.30 ± 13.12	65.00	62.92 ± 12.27	***p*** < 0.001*	**U = 237.000**
p value and Test Statisticᵈ	***p*** < 0.001* Z: -4.953		0.874 Z: -0.158			
Pretest Application; Social Functioning Subscaleᵇ	75.00	69.46 ± 23.03	75.00	73.33 ± 15.02	0.548	U = 612.000
Posttest Application; Social Functioning Subscaleᵇ	90.00	86.35 ± 14.46	72.50	70.14 ± 18.46	***p*** < 0.001*	**U = 325.500**
*p value and Test Statistic*ᵈ	***p*** < 0.001* Z:-3.894		0.282 Z:-1.075			
Pretest Application; School Functioning Subscaleᵃ	55.00	56.62 ± 17.48	60.00	57.50 ± 14.37	0.816	t: 0.234
Posttest Application; School Functioning Subscaleᵇ	75.00	70.95 ± 17.51	57.50	54.86 ± 11.80	***p*** < 0.001*	**U = 278.500**
p-value and Test Statisticᵈ	***p*** < 0.001* Z: -3.661		0.188 Z: -1.317			
Pretest Application; Psycho-Social Health Subscaleᵃ	63.33	62.57 ± 15.83	64.33	64.21 ± 11.36	0.611	t: 0.511
Posttest Application; Psycho-Social Health Subscaleᵃ	81.67	79.86 ± 12.07	62.50	62.64 ± 11.49	***p*** < 0.001*	**t: 3.241**
*p value and Test Statistic*ᵃ	***p*** < 0.001* t:-7.299		0.303 t: 1.045			
Pretest Application; Total Score of PedsQLᵃ	65.00	66.25 ± 15.67	59.69	60.61 ± 12.16	0.620	t: 0.497
Posttest Application; Total Score of PedsQLᵇ	80.00	80.47 ± 11.95	61.88	62.71 ± 11.19	***p*** < 0.001*	**U = 202.500**
p value and Test Statisticᵈ	***p*** < 0.001* Z:-5.137		0.781 Z: -0.278			

^*a*^ *Independent samples t test*

^*b*^ *Mann‒Whitney U test*

^*c*^ *Paired t test*

^*d*^ *Wilcoxon test*

*α = 0.05; Significant difference between groups*

Table 3 presents the between-group and within-group comparisons of the pretest and posttest subscale scores and total mean scores of the RCADS-CV for children with ADHD. Statistical analyses were performed based on T scores (Table 3).

**Table 3:** Comparison of Pre- and posttest scores on the revised anxiety and depression Scale – Children’s version in study groups

T Scores of the RCADS-CV Subscales and Total (Pre-Test and Post-Test Applications)	Intervention Group		Control Group			
	Median	Ort ± Ss	Median	Median	Ort ± Ss	Median
Pretest Application; Separation Anxiety Subscaleᵇ	71.00	69.76 ± 9.72	75.50	72.50 ± 8.79	0.206	U:553.500
Posttest Application; Separation Anxiety Subscaleᵇ	55.00	54.97 ± 8.93	74.00	70.17 ± 11.17	***p*** < 0.001*	**U:208.000**
*p value and Test Statistic*ᵈ	***p*** < 0.001* Z:-4.967		0.085 Z:-1.720			
Pretest Application; Social Phobia Subscaleᵃ	60.00	58.27 ± 8.57	59.00	57.22 ± 9.69	0.654	t:0.489
Posttest Application; Social Phobia Subscaleᵇ	40.00	40.27 ± 8.23	57.00	55.78 ± 9.96	***p*** < 0.001*	**U:164.500**
p value and Test Statisticᵈ	***p*** < 0.001* Z:-4.989		0.734 Z: -0.340			
Pretest Application; Obsessive-Compulsive Disorder Subscaleᵇ	59.00	58.35 ± 12.71	54.50	53.92 ± 10.43	0.232	U:472.000
Posttest Application; Obsessive-Compulsive Disorder Subscaleᵃ	41.00	42.49 ± 7.25	55.00	56.00 ± 12.35	0.003*	**t:-5.720**
p value and Test Statisticᵈ	***p*** < 0.001* Z:-4.679		0.090 Z:-1.694			
Pretest Application; Panic Disorder Subscaleᵃ	63.00	61.97 ± 11.19	60.00	61.03 ± 13.25	0.228	t:-0.329
Posttest Application; Panic Disorder Subscaleᵇ	45.00	46.27 ± 8.59	63.00	61.53 ± 14.09	***p*** < 0.001*	**U:237.000**
p value and Test Statisticᵈ	***P*** < 0.001* Z:-4.958		0.555 Z: -0.591			
Pretest Application; Generalized Anxiety Disorder Subscaleᵃ	55.00	54.57 ± 8.83	56.00	55.00 ± 8.78	0.877	t: -1.264
Posttest Application; Generalized Anxiety Disorder Subscaleᵃ	39.00	39.62 ± 7.03	56.50	55.08 ± 11.02	0.001*	**t:-7.168**
p value and Test Statisticᶜ	***p*** < 0.001* t:9.171		0.961 t:-0.49			
Pretest Application; Major Depressive Disorder Subscaleᵃ	56.00	57.35 ± 13.29	56.50	57.69 ± 13.60	0.918	t: 0.109
Posttest Application; Major Depressive Disorder Subscaleᵇ	40.00	39.89 ± 9.79	59.50	57.28 ± 15.47	***p*** < 0.001*	**U:254.500**
p value and Test Statisticᵈ	***p*** < 0.001* Z:-5.130		0.620 Z: -0.496			
Pretest Application; Total Anxiety Disorder Scoreᵇ	66.00	63.27 ± 7.46	62.00	61.64 ± 11.53	0.663	U:626.500
Posttest Application; Total Anxiety Disorder Scoreᵇ	41.00	41.78 ± 6.69	65.00	61.14 ± 13.69	***p*** < 0.001*	**U:162.000**
p value and Test Statisticᵈ	***p*** < 0.001* Z:-5.305		0.701 Z: -0.385			
Pretest Application; Total Depression-Anxiety Disorder Scoreᵃ	65.00	63.43 ± 8.45	63.00	61.83 ± 12.42	0.113	t: 0.720
Posttest Application; Total Depression-Anxiety Disorder Scoreᵇ	40.00	39.86 ± 9.29	63.50	61.17 ± 14.56	***p*** < 0.001*	**U:166.000**
p value and Test Statisticᵈ	***p*** < 0.001* Z:-3.506		0.822 Z: -0.226			

^*a*^ *Independent samples t test*

^*b*^ *Mann‒Whitney U test*

^*c*^ *Paired t test*

^*d*^ *Wilcoxon test*

*α = 0.05; Significant difference between groups*

The study revealed no significant differences between the intervention and control groups in the pretest scores of the RCADS-CV total scores and subscales. However, significant improvements were observed in the posttest scores of the intervention group. Specifically, significant improvements in separation anxiety (*p* < 0.001, U = 208.000), social phobia (*p* < 0.001, U = 164.500), obsessive-compulsive disorder (*p* = 0.003, t = -5.720), panic disorder (*p* < 0.001, U = 237.000), generalized anxiety disorder (*p* = 0.001, t = -7.168), major depressive disorder (*p* < 0.001, U = 254.500), total anxiety disorder score (*p* < 0.001, U = 162.000), and total depression‒anxiety disorder score (*p* < 0.001, U = 162.000) were noted in the intervention group compared with the control group posttest scores (*p* < 0.001). Within-group analyses also revealed significant differences in the intervention group (*p* < 0.001), whereas no significant differences were detected in the control group (*p* > 0.05). In conclusion, marked posttreatment improvements were observed in the intervention group, whereas no changes were observed in the control group (Table 3).

Table 4 presents the between-group and within-group comparisons of the pretest and posttest mean scores on the subscales and total scores of the RCADS-PV, as completed by the parents of children with ADHD. Statistical analyses were conducted on the basis of the T scores (Table 4).

**Table 4:** Comparison of Pre- and posttest scores on the revised anxiety and depression Scale – Parent version in study groups

T Scores of the RCADS-PV Subscales and Total (Pre-Test and Post-Test Applications)	Intervention Group		Control Group			
	Median	Ort ± Ss	Median	Median	Ort ± Ss	Median
Pretest Application; Separation Anxiety Subscaleᵇ	80.00	74.92 ± 8.88	80.00	71.72 ± 11.86	0.422	U:600.500
Posttest Application; Separation Anxiety Subscaleᵇ	62.00	63.11 ± 9.93	80.00	71.56 ± 12.03	0.001*	**U:348.000**
*p value and Test Statistic*ᵈ	***p*** < 0.001* Z:-4.072		0.924 Z: -0.095			
Pretest Application; Social Phobia Subscaleᵃ	63.00	62.73 ± 9.19	63.00	61.75 ± 12.18	0.550	t:0.387
Posttest Application; Social Phobia Subscaleᵇ	47.00	47.41 ± 6.32	64.00	62.03 ± 13.22	***p*** < 0.001*	**U:244.000**
p value and Test Statisticᵈ	***p*** < 0.001* Z:-4.952		0.977 Z:-0.029			
Pretest Application; Obsessive-Compulsive Disorder Subscaleᵇ	61.00	63.49 ± 10.43	61.50	64.28 ± 12.49	0.846	U:648.500
Posttest Application; Obsessive-Compulsive Disorder Subscaleᵃ	51.00	53.24 ± 8.91	62.00	62.28 ± 13.35	0.003*	**U:399.000**
p value and Test Statisticᵈ	***p*** < 0.001* Z:-4.483		**0.043 Z:-2.206**			
Pretest Application; Panic Disorder Subscaleᵃ	65.00	66.84 ± 10.62	79.50	69.42 ± 13.76	0.139	U:535.000
Posttest Application; Panic Disorder Subscaleᵇ	53.00	57.19 ± 10.10	75.50	67.92 ± 13.67	0.001*	**U:360.000**
p value and Test Statisticᵈ	***p*** < 0.001* Z:-3.439		0.304 Z:-1.027			
Pretest Application; Generalized Anxiety Disorder Subscaleᵃ	58.00	60.46 ± 7.14	64.50	64.33 ± 11.54	0.131	U:529.500
Posttest Application; Generalized Anxiety Disorder Subscaleᵃ	51.00	50.70 ± 6.67	61.00	63.31 ± 11.17	***p*** < 0.001*	**U:383.500**
p value and Test Statisticᶜ	***p*** < 0.001* t:7.105		p: 0.465 t:0.739			
Pretest Application; Major Depressive Disorder Subscaleᵃ	63.00	62.95 ± 9.30	63.50	61.69 ± 17.80	0.778	U:640.500
Posttest Application; Major Depressive Disorder Subscaleᵇ	50.00	50.59 ± 7.48	66.00	62.83 ± 14.94	***p*** < 0.001*	**U:341.000**
p value and Test Statisticᵈ	***p*** < 0.001* Z:-4.761		0.751 Z:-0.318			
Pretest Application; Total Anxiety Disorder Scoreᵇ	72.00	70.14 ± 8.42	74.50	68.75 ± 13.05	0.712	U:633.000
Posttest Application; Total Anxiety Disorder Scoreᵇ	52.00	53.57 ± 7.36	72.50	67.36 ± 13.78	***p*** < 0.001*	U:286.500
p value and Test Statisticᵈ	***p*** < 0.001* Z:-5.096		0.084 Z:-1.730			
Pretest Application; Total Depression-Anxiety Disorder Scoreᵃ	71.00	69.81 ± 8.24	75.00	68.25 ± 13.75	0.540	U:611.000
Posttest Application; Total Depression-Anxiety Disorder Scoreᵇ	53.00	52.70 ± 7.51	69.50	63.14 ± 19.15	0.001*	**U:362.000**
p value and Test Statisticᵈ	***p*** < 0.001* Z:-5.163		0.065 Z:-1.845			

^*a*^ *Independent samples t test*

^*b*^ *Mann‒Whitney U test *

^*c*^ *Paired t test*

^*d*^ *Wilcoxon test*

*α = 0.05; Significant difference between groups*

According to the T score analyses of the posttest data, significant improvements in the following subscales and total scores were observed in the intervention group compared with the control group: separation anxiety (*p* < 0.001, U = 348.000), social phobia (*p* < 0.001, U = 244.000), obsessive‒compulsive disorder (*p* = 0.003, U = 399.000), panic disorder (*p* = 0.001, U = 360.000), generalized anxiety disorder (*p* < 0.001, U = 383.500), major depressive disorder (*p* < 0.001, U = 341.000), total anxiety disorder score (*p* < 0.001, U = 286.500), and total depression‒anxiety disorder score (*p* = 0.001, U = 362.000). These findings indicate a statistically significant improvement in the intervention group’s posttest scores.

Within-group analyses revealed statistically significant differences between the pretest and posttest scores in the intervention group (*p* < 0.001), whereas no significant differences were found in the control group (*p* > 0.05). In conclusion, the intervention group showed marked improvements following the treatment, whereas no notable changes were observed in the control group (Table 4).

## Discussion

This study aimed to investigate the effects of a CBT-based psychoeducation program on the quality of life, anxiety, and depression levels in children with ADHD. Specifically, the study tested two hypotheses: H1, that the program would improve the quality of life of children with ADHD, and H2, that it would reduce their anxiety and depression levels. Our findings indicate that the intervention produced significant improvements in both quality of life and emotional symptoms. Children in the intervention group showed significant increases in total PedsQL scores and in the subscales of physical health, emotional functioning, social functioning, school functioning, and psychosocial health compared to pretest measurements. These improvements were statistically significant compared to the control group and clinically meaningful, reflecting enhanced daily functioning, improved emotional regulation skills, and positive changes in family interactions. This suggests that the CBT-based psychoeducation program not only targets symptom reduction but also contributes to children’s overall adaptation in daily life, likely through CBT’s structured approach fostering self-awareness, behavioral regulation, and adaptive strategies that generalize across academic and social contexts. Clinically, these improvements may manifest as better school performance, enhanced peer relationships, and smoother family interactions.

The literature indicates that children with ADHD generally experience reduced quality of life, particularly in school and emotional functioning domains, alongside impairments in social functioning and overall psychosocial health [13, 31–37]. Clinically, improvements in these areas correspond to increased stability in classroom performance, reduced emotional outbursts, and smoother interactions with peers and family. This supports the theoretical premise that ADHD-related executive function deficits—such as attentional regulation and impulse control—may indirectly reduce quality of life, and that CBT-based interventions may address these mechanisms through cognitive restructuring and behavioral rehearsal. Previous studies have demonstrated that CBT-based psychoeducation effectively enhances social skills and school functioning in children with ADHD [13, 31–38]. Kavurma (2023) reported significant improvements in quality of life following social skills training [39], while peer-involved interventions were also shown to enhance social functioning and positively impact quality of life [40, 41]. A systematic review by Ojinna et al. (2022) highlighted that both CBT and methylphenidate treatments reduce symptoms and improve quality of life in children with ADHD [40, 42]. Consistent with this literature, our findings suggest that CBT facilitates social integration and emotional adjustment beyond symptom control through behavioral and cognitive restructuring, which clinically translates into reductions in anxiety and depression, increased classroom participation, and more harmonious interactions at home.

Post-intervention, significant reductions were observed in anxiety and depression symptoms, including subscales of separation anxiety, social phobia, obsessive-compulsive disorder, panic disorder, generalized anxiety disorder, and major depression (*p* < 0.001). Total anxiety and depression scores decreased significantly only in the intervention group, indicating the efficacy of the program. These effects likely occurred through mechanisms such as enhancing emotional regulation, restructuring maladaptive cognitions, and developing problem-solving skills. Literature shows that children with ADHD exhibit higher levels of depression and anxiety compared to neurotypical peers, often with frequent comorbid psychiatric diagnoses [22, 43–46]. Karaman et al. (2013) reported elevated anxiety and depression in children aged 7–12 with ADHD, and Koyuncu et al. (2018) noted high prevalence rates for general and specific anxiety disorders in this population [45, 46]. These findings underscore the necessity of addressing comorbid internalizing symptoms in clinical interventions.

The CBT-based psychoeducation program likely mitigated risk factors and prevented internalizing symptoms by teaching social communication and emotional self-regulation strategies. While pharmacological treatments are commonly used, non-pharmacological approaches are essential [47]. CBT-based interventions delivered by nurses have been reported to enhance problem-solving skills, peer relationships, parenting practices, emotional regulation, self-esteem, and behavioral responses [21, 48]. Numerous studies emphasize the efficacy of CBT in treating anxiety and major depression, demonstrating that cognitive-behavioral interventions modify maladaptive cognitions and support adaptive thoughts and coping behaviors [49–53]. CBT, often combined with medication, is considered a valuable complementary intervention for reducing comorbid symptoms in children with ADHD [49, 50]. Antshel et al. (2014) and Sibley et al. (2016) reported significant reductions in anxiety and depression symptoms following CBT, while Kendall et al. (2008) and Ginsburg et al. (2012) observed improvements in social phobia, generalized anxiety, separation anxiety, and panic disorder [49–53]. Asutay and Karaaziz (2023) and Coelho et al. (2017) reported improvements in ADHD symptoms and overall adjustment post-CBT [54, 55]. In this study, components of emotional regulation, problem-solving, and impulse control likely contributed to reductions in anxiety and depression, supporting the theoretical model in which emotional dysregulation mediates the relationship between ADHD symptoms and internalizing disorders, and can be addressed through CBT [54, 55].

While the majority of findings are consistent with the literature, some studies report no significant changes in quality of life or internalizing symptoms, particularly in adolescents and young adults [10, 37, 38]. This variability may be explained by developmental stage, age, cognitive maturity, environmental demands, motivation, comorbid conditions, and intervention design. These findings highlight the importance of tailoring psychoeducation programs to individual developmental and contextual needs.

From a nursing perspective, the results emphasize the theoretical and clinical importance of interventions that support children’s psychosocial development and quality of life. Nurses can play an active role in multidisciplinary teams by implementing psychoeducation programs, facilitating social skills development, informing families, and contributing to early diagnosis and intervention processes. These findings reinforce the holistic health approach in nursing, encompassing physical, emotional, and social well-being within a biopsychosocial model [9, 12, 38]. Clinically, providing psychosocial support, educating families, and offering guidance—beyond symptom monitoring—constitute a comprehensive approach.

In conclusion, CBT-based psychoeducation programs can be considered effective nursing interventions for reducing anxiety and depression symptoms and improving quality of life in children with ADHD. By translating CBT mechanisms into practical interventions, nurses can enhance emotional regulation, problem-solving, and impulse control, promoting both mental health and developmental resilience through cognitive restructuring and behavioral modeling.

### Limitations and recommendations

This study has several limitations that should be considered when interpreting the findings. First, it was conducted at a single center with a relatively small sample size, which may limit the generalizability of the results. The limited sample and single-center design may have restricted the diversity of clinical and demographic characteristics, potentially affecting the stability of estimates and the precision of effect size calculations. Missing data were addressed using listwise deletion, which, given the small sample, may have further reduced statistical power and led to underestimation of intervention effects.

Second, the intervention consisted of only eight sessions, and posttest assessments were conducted at slightly different time points for the intervention and control groups. The short-term evaluation and absence of follow-up measurements prevent conclusions about the durability of treatment effects.

Third, the study design did not allow for blinding of practitioners or evaluators, and the same researcher conducted both the intervention and data collection, introducing potential researcher bias. While using both child and parent reports strengthens methodological rigor, self-reported data remain susceptible to response bias.

Future research should prioritize larger, multicenter studies to enhance generalizability and population diversity. Longitudinal follow-up assessments are needed to determine the persistence of treatment gains, and studies should examine the specific impact of parental involvement. Implementing blinding procedures and separating intervention delivery from outcome assessment can minimize potential bias.

## Conclusion

This study demonstrates that a CBT-based psychoeducation program significantly improved the quality of life and reduced anxiety and depression symptoms in children with ADHD. Improvements were observed across multiple dimensions—including physical, emotional, social, and school functioning—as reported by both children and their parents. No significant changes were detected in the control group, supporting the efficacy of the intervention.

These findings align with previous research showing that CBT interventions enhance psychosocial functioning, emotional regulation, and adaptive coping strategies in children with ADHD [31–36, 39–42, 49–53]. The program’s structured approach likely facilitated improvements in behavioral regulation, social skills, and problem-solving abilities, contributing to clinically meaningful gains in daily functioning.

The results highlight the critical role of nurses in implementing psychoeducation-based interventions. By providing holistic care that addresses both psychosocial and emotional needs, nurses can enhance intervention outcomes. Involving and educating parents further strengthens these benefits and supports sustainable improvements in children’s mental health and daily functioning.

Integrating CBT-based psychoeducation programs into routine nursing practice and expanding nurse training on these interventions are recommended to improve care for children with ADHD. Future research should focus on long-term, multicenter studies to confirm the durability and generalizability of these effects.

## Supplementary Information

Supplementary Material 1.

## Data Availability

The datasets generated and/or analyzed during the current study are not publicly available due to legal regulations concerning the protection of personal data. However, they are available from the corresponding author on reasonable request.

## Abbreviations

ADHD: Attention-Deficit/Hyperactivity Disorder
CBT: Cognitive Behavioral Therapy
RCADS-PV: Revised Child Anxiety and Depression Scale – Parent Version
RCADS-CV: Revised Child Anxiety and Depression Scale – Child Version
PedsQL: Pediatric Quality of Life Inventory
