# Supplementary material for: The effects of cognitive behavioral therapy-based psychoeducation on quality of life, anxiety, and depression in children with attention deficit hyperactivity disorder
Source: BMC Psychol. 2026 Feb 7;14:342. doi: 10.1186/s40359-026-04110-7 (PMC12977643; doi:10.1186/s40359-026-04110-7)
Supplement: Supplementary file 1 — Supplementary Material 1. [file 40359_2026_4110_MOESM1_ESM.docx]

**Cognitive-Behavioral Therapy-Based Psychoeducation Session Contents**

**Sessıons**

1. Session: Introduction, Providing Information to the Child and Family about CBT-Based Psychoeducation
2. Session: General Information about ADHD
3. Session: Recognizing Our Emotions
4. Session: Problem-Solving, Understanding Problems Caused by ADHD, Awareness of Impulsivity and Hyperactivity
5. Session: Training to Inhibit Impulsivity
6. Session: Attention-Focusing Training
7. Session: Finding Appropriate Behavior
8. Session: Conducting General Evaluation and Closure

**The tools and psychoeducational techniques used in the CBT-Based Psychoeducational Program are listed below;**

- **Emotion Cards** are an effective tool used to help children recognize, express, and understand their emotions. Particularly in psychoeducation programs, emotion cards can strengthen communication with children by providing a play-based approach.
- **Exercise sheets** were prepared for each session to allow participants to follow the instructions described below.
- The **ABC model** was utilized to help children become aware of their core, automatic thoughts and negative beliefs. This model, used in CBT, aids in understanding how our emotional responses and behaviors are shaped. It clearly demonstrates that an individual’s reactions to events are influenced by their thought processes (Artıran & Şeker, 2020). In the ABC model: A represents a situation or event that triggers an emotional response; B represents the thoughts, interpretations, or beliefs the individual holds regarding the event; C represents the emotional or behavioral responses resulting from those thoughts.
- As part of the educational content, **magnifying glasses** were provided to each child to support attention and focus during shape and puzzle activities.

**Session 1: Introduction, Psychoeducation on CBT for Children and Parents**

**Objective:**

- To ensure that the child and parent participating in the CBT-based psychoeducation program understand the program’s objectives and rules.
- To introduce the CBT-based psychoeducation program and administer the pre-test.

**Techniques Used:**

- Lecture
- Question-and-answer
- Use of visual materials

**Expected Outcomes:**

- The child and parent will gain knowledge about the CBT-based psychoeducation program and be able to adapt to it.

**Session Content and Procedures:**

- The therapist introduces themselves.
- The child and parents are informed about CBT and the structure of the program.
- Administration of the pre-test.
- Explanation of the objectives and rules of the therapy process.
- Identification of shared and individual goals to be achieved at the end of the therapy.
- Collection of contact information.
- Planning the time for the next session.

**Summary of Session:**

The researcher began the session by introducing themselves to the participating child and parent. The content of the CBT-based psychoeducation was presented. It was explained that the program would consist of a total of eight sessions, with different topics covered in each session. Key activities for both the child and the parent, as well as homework assignments, were described.

The reward system, which reinforces the principles of incentive-based learning central to CBT interventions, was explained. The child would start each session with 10 points; by following instructions correctly and adhering to the rules, points could be used to obtain rewards from a “reward market” (including items such as toys, balls, sports jerseys, cinema tickets, meals, etc.) chosen together with the parent. Points would be deducted for any mistakes. It was emphasized that all sessions would involve both the child and the parent participating together. The importance of continuity for the effective implementation of the program was highlighted. Expected changes following the intervention, as well as the expectations of both the child and parent from therapy, were discussed and goals were established.

Following the administration of the pre-test, planning for the next session was carried out, contact information was collected, and the session was concluded.

**Session 2: General Information on ADHD**

**Objective:**

- To provide the child and parent with information about ADHD within the scope of the program.

**Techniques Used:**

- Lecture
- Question-and-answer
- Use of visual materials
- Discussion

**Expected Outcomes:**

- The child and parent will be able to identify ADHD symptoms and understand how these symptoms affect their daily life.

**Session Content and Procedures:**

- Evaluation of the previous session.
- Providing information to the child and parent about ADHD, including:
  - Diagnosis
  - Symptoms
  - Types and prevalence
  - General information about treatment
- Planning the time for the next session

**Summary of Session:**

The previous session was reviewed with the child and parent, and any questions related to the session were addressed. The purpose of this session was to provide general information about ADHD. Information regarding ADHD symptoms, diagnosis, treatment, prevalence, and etiology was provided, and participants’ questions were answered.

**Session 3: Recognizing Our Emotions**

**Objectives:**

- To help children recognize the emotions of individuals in their environment and adjust their behavior accordingly.
- To teach children strategies to cope with increased stress and emotional intensity due to ADHD symptoms.

**Techniques Used:**

- Lecture
- Question-and-answer
- Visual materials
- Role-play using the ABC model
- Emotion cards

**Expected Outcomes:**

- The child will be able to recognize their own emotions and express them appropriately.

**Session Content and Procedures:**

- Evaluation of the previous session
- Explanation of the relationship between thoughts, emotions, and behaviors using the ABC model of CBT
- Identification of core, intermediate, and automatic thoughts
- Activities with the child to recognize different emotions in their environment
- Identifying behavioral changes that arise from different emotions

**Homework:**

1. Complete a visual form related to different emotions.
2. Bring three different pictures from any magazine or newspaper and identify the emotions of the people depicted.

**Summary of Session:**

In order to modify behavior and resolve interpersonal problems, children need to recognize the emotions arising in interactions. To enable the child to respond appropriately and correctly to different emotional states, the ABC model of CBT was explained. Within this framework, the concept of “emotion” was defined. Different emotions, such as anger, frustration, happiness, shame, and fear, were introduced using visual emojis. Using emotion worksheets provided by the therapist, the child was asked to write down emotions that make them feel good and those that make them feel bad.

In the ABC model, A represents the event the individual encounters; B represents the individual’s perception and interpretation of the event, including positive, neutral, or negative thoughts; and C represents the emotional, behavioral, and physiological responses resulting from this interpretation. Examples were provided to help the child identify the appropriate behavioral response to different situations according to the ABC model. Homework assignments were given, the next session was planned, and Session 3 was concluded.

**Session 4: Problem-Solving – Understanding ADHD-Related Problems, Impulsivity and Hyperactivity Awareness, Self-Regulation**

**Objectives:**

- To recognize ADHD symptoms and their effects on behavior and emotional states.
- To identify and understand impulsive behaviors and hyperactivity arising from ADHD.

**Techniques Used:**

- Worksheets incorporating the five-step problem-solving method
- Exercises: alphabet completion, number sequencing, identifying geometric shapes
- “From vocalization to internalization” method

**Expected Outcomes:**

- The child can apply step-by-step problem-solving strategies to develop solutions for encountered problems.

**Session Content and Procedures:**

- Review of the previous session
- Homework review
- Discussing current issues to create a session agenda
- Teaching the child problem-solving steps for issues arising from ADHD-related impulsivity, hyperactivity, and attention difficulties

**Homework:**

1. Apply the problem-solving steps to address issues experienced at home and at school.
2. Repeat the problem-solving steps from vocalization to silent internalization while completing assigned tasks.

**Summary of Session:**

The previous session was reviewed, and homework was checked. A five-minute discussion was conducted with participants to identify current problems and create a session agenda. ADHD-related issues affecting problem-solving, such as impulsivity, distractibility, and hyperactivity, were discussed.

**The five-step problem-solving strategy was explained:**

1. **“**What do I need to do?” – defining the problem clearly.
2. “Have I considered all options?” – evaluating all possible solutions.
3. “Focus” – ensuring attention on the options.
4. “Check your answers” – review all potential solutions.
5. “Well done!” – self-praise after choosing the correct solution.

Example exercises emphasized accuracy and focus over speed. Exercises such as alphabet completion, number sequencing, and geometric shape identification were conducted, initially aloud, then whispered, and finally silently. This method aimed to teach the child self-guided problem-solving strategies, reinforcing behavioral change (Kendall & Braswell, 1982). The next session was planned, homework was assigned, and the session concluded.

**Session 5: Training to Inhibit Impulsivity**

**Objectives:**

- To improve the child’s sustained attention in social situations for effective problem-solving.
- To encourage the child to proceed step-by-step in problem-solving rather than responding impulsively.

**Techniques Used:**

- Question-and-answer
- Discussion
- Worksheets focused on solving specific problems

**Expected Outcomes:**

- The child can use appropriate verbal and non-verbal communication with peers and others.
- The child can learn cooperative skills during group activities**.**

**Session Content and Procedures:**

- Review of the previous session and recall of problem-solving steps
- Homework review
- Discuss current issues to create a session agenda
- Identify negative emotions
- Increase awareness of impulses
- Learn strategies to prevent negative behaviors (behavioral reversal, attention shifting, physical exercises, etc.)
- Continue practice with example problem-solving exercises

**Homework:**

- Record negative situations encountered before the next session and note which technique was applied to resolve them.

**Summary of Session:**

The previous session was reviewed, and homework was checked. Five-minute discussions were held with participants to create a session agenda. The five-step problem-solving method from the previous session was repeated with example exercises. The child applied problem-solving strategies to issues experienced at home and school. Initially, the steps were verbalized aloud, then whispered, and finally repeated silently, reinforcing self-guided problem-solving skills.

The next session was planned, homework was assigned, and the session concluded**.**

**Session 6: Attention Training**

**Objectives:**

- To help the child sustain attention longer for effective problem-solving.
- To improve attention span to positively impact academic performance.
- To encourage step-by-step problem-solving instead of impulsive responses.
- To teach the child the sequential steps required to solve a specific problem and ensure they follow these steps accurately.
- To help the child adhere to instructions and complete steps correctly during problem-solving.

**Techniques Used:**

- Word-finding game with three difficulty levels: easy, medium, hard
- Magnifying glass to ensure careful observation

**Expected Outcomes:**

- The child can plan and follow through with daily tasks and activities.
- The child can prioritize tasks and manage time effectively.
- The child can develop step-by-step plans to complete homework and projects on time.

**Session Content and Procedures:**

- Review of the previous session
- Recall of problem-solving steps
- Homework review
- Discuss current issues to create a session agenda
- Emphasis on organized, structured, and focused problem-solving rather than random trial-and-error approaches
- During exercises, problem-solving steps were repeated from vocalization to silent internalization

**Homework:**

- Complete the word-finding game assigned by the researcher, maintaining careful attention until the next session.

**Summary of Session:**

The previous session was reviewed, and homework was checked. A five-minute discussion with participants was conducted to create a session agenda. The word-finding game was implemented, instructing the children to locate words carefully rather than rushing or guessing. Magnifying glasses were provided to slow down progress and enhance focus. Children were instructed to focus only on one section of the puzzle at a time to improve attention.

The next session was planned, homework was assigned, and the session concluded**.**

**Session 7: Identifying Appropriate Behavior**

**Objectives:**

- The child should be able to clearly identify and define problems.
- The child should generate multiple potential solutions and evaluate the advantages and disadvantages of each.
- The child should consider the possible outcomes of each solution and assess their emotional and social effects on themselves and others, demonstrating empathy and selecting the most appropriate behavior.

**Techniques Used:**

- Questioning
- Discussion
- Role-playing

**Expected Outcomes:**

- The child can develop self-confidence by recognizing strengths and achievements.
- The child can acknowledge mistakes and learn from them.
- The child can generate alternative solutions to problems encountered.

**Session Content and Procedures:**

- Review of the previous session and recall of problem-solving steps
- Homework review
- Discuss current issues to create a session agenda
- Problem-solving steps for encountering a challenge:
  1. What is the problem here?
  2. What can you do to solve the problem?
  3. Focus on potential outcomes and ask: “How would I feel? How would the other person feel? What would the other person do based on my choice?” to select the most appropriate behavior
- Develop coping strategies for behaviors arising from emotional responses to problems
- Scenario-based discussion to identify potential behaviors and select the most suitable one (Kendall, 2011)

**Homework:**

- Select a problem encountered at school, evaluate at least three possible solutions, and choose the most appropriate one.

**Summary of Session:**

The previous session was reviewed, and homework was checked. Five-minute discussions with participants were conducted to create a session agenda. Situation cards describing potential real-life and school problems were distributed. Using problem-solving steps, children applied the “What if?” method to select the most appropriate behavior. Initially, steps were verbalized aloud and later repeated silently during scenario exercises.

The next session was planned, homework was assigned, and the session concluded.

**Session 8: General Evaluation and Closure**

**Objectives:**

- To evaluate the CBT-based psychoeducation program and collect final data.

**Expected Outcomes:**

- Objective assessment of the CBT-based psychoeducation using applied tests.
- Increased awareness of self for both child and parent.

**Session Content and Procedures:**

- Review of the previous session
- Homework review
- Reinforcement of previously learned skills
- Self-assessment by child and parent
- Address any remaining gaps in the program
- Overall session evaluation and feedback collection
- Administration of final test

**Summary of Session:**

The previous session was reviewed, and homework was checked. Problem-solving steps learned in previous sessions were reinforced. Children and parents compared past and current performance and self-awareness. Remaining cognitive or behavioral areas not addressed in prior sessions were evaluated. Feedback was collected regarding the instructor. Final tests were administered, data collected, and the session concluded.

**References:**

Artıran, M., & Şeker, A. (2020). The predictive role of automatic thoughts: Differences between hope and hopelessness. *Elektronik Sosyal Bilimler Dergisi, 19*(73), 36–47. <https://doi.org/10.17755/esosder.488733>

Kendall, P. C., & Braswell, L. (1982). Cognitive-behavioral self-control therapy for children: A components analysis. *Journal of Consulting and Clinical Psychology, 50*(5), 672–689. <https://doi.org/10.1037/0022-006X.50.5.672>

Kendall, P. C. (2011). *Cognitive-behavioral therapy for impulsivity and ADHD: Therapist implementation guide – Stop and Think workbook* (V. Görmez & A. Yılmaz, Trans., 2nd ed., pp. 24–36). Apamer Publishing.
